# Perspectives and Issues in the Assessment of *SMARCA4* Deficiency in the Management of Lung Cancer Patients

**DOI:** 10.3390/cells10081920

**Published:** 2021-07-29

**Authors:** Subasri Armon, Paul Hofman, Marius Ilié

**Affiliations:** 1 Laboratory of Clinical and Experimental Pathology, Hospital-Integrated Biobank (BB-0033-00025), Nice Hospital University, FHU OncoAge, Université Côte d’ Azur, 06000 Nice, France; sarmon2003@gmail.com (S.A.); hofman.p@chu-nice.fr (P.H.); 2 Histopathology Unit, Department of Pathology, Hospital Kuala Lumpur, Jalan Pahang, Kuala Lumpur 50586, Malaysia

**Keywords:** non-small cell lung carcinoma, *SMARCA4* gene, solid adenocarcinoma, immunohistochemistry, sequencing

## Abstract

Lung cancers are ranked third among the cancer incidence in France in the year 2020, with adenocarcinomas being the commonest sub-type out of ~85% of non-small cell lung carcinomas. The constant evolution of molecular genotyping, which is used for the management of lung adenocarcinomas, has led to the current focus on tumor suppressor genes, specifically the loss of function mutation in the *SMARCA4* gene. *SMARCA4*-deficient adenocarcinomas are preponderant in younger aged male smokers with a predominant solid morphology. The importance of identifying *SMARCA4*-deficient adenocarcinomas has gained interest for lung cancer management due to its aggressive behavior at diagnosis with vascular invasion and metastasis to the pleura seen upon presentation in most cases. These patients have poor clinical outcome with short overall survival rates, regardless of the stage of disease. The detection of *SMARCA4* deficiency is possible in most pathology labs with the advent of sensitive and specific immunohistochemical antibodies. The gene mutations can be detected together with other established lung cancer molecular markers based on the current next generation sequencing panels. Sequencing will also allow the identification of associated gene mutations, notably *KRAS*, *KEAP1*, and *STK11,* which have an impact on the overall survival and progression-free survival of the patients. Predictive data on the treatment with anti-PD-L1 are currently uncertain in this high tumor mutational burden cancer, which warrants more groundwork. Identification of target drugs is also still in pre-clinical testing. Thus, it is paramount to identify the *SMARCA4*-deficient adenocarcinoma, as it carries worse repercussions on patient survival, despite having an exceptionally low prevalence. Herein, we discuss the pathophysiology of *SMARCA4*, the clinicopathological consequences, and different detection methods, highlighting the perspectives and challenges in the assessment of *SMARCA4* deficiency for the management of non-small cell lung cancer patients. This is imperative, as the contemporary shift on identifying biomarkers associated with tumor suppressor genes such as *SMARCA4* are trending; hence, awareness of pathologists and clinicians is needed for the *SMARCA4*-dNSCLC entity with close follow-up on new management strategies to overcome the poor possibilities of survival in such patients.

## 1. Introduction

Lung cancer in France is the third most common cancer (10.3%) diagnosed among its 65 million population, and it is the second most prevalent cancer worldwide (11.4%), having claimed 1,796,144 lives in the year 2020 [1]. It is the leading cause of premature deaths in France [2]. It is common knowledge that lung cancer is subdivided into small cell lung carcinoma (SCLC; 15%) and non-small cell lung carcinoma (NSCLC; 85%), of which 40% of NSCLCs are lung adenocarcinomas [3]. Most of these cases are advanced and have metastasis at the time of diagnosis, bringing down the average 5-year survival rate to ~15%, despite the advances in molecular profiling and personalized medicine [3].

According to the National Cancer Institute, U.S.A. there is a paradigm shift in the treatment of advanced NSCLC in the past decade. The identification of targeted therapy based on “druggable” oncogenes have provided new light at the end of the tunnel for patients who harbor driver mutations in relation to survival and morbidity rates of the disease and treatment, respectively. In addition, with the advent of immune-based therapy namely, programmed cell death protein 1 and programmed death ligand 1 (PD-1 and PD-L1, respectively) inhibitors, improvements in NSCLC treatment outcomes are tremendous. The latest version of the National Comprehensive Cancer Network Guidelines for NSCLC has recommended a few evidence-based predictive biomarkers for adenocarcinomas, large cell carcinomas, and NSCLC-NOS, in particular *EGFR* mutations, *ALK* rearrangements, *ROS1* rearrangements, *BRAF* V600E mutation, *KRAS* mutations, *NTRK* 1/2/3 gene fusion, *METex14* skipping mutations, *RET* rearrangements, and PD-L1 expression. The advice was to conduct these tests as broad molecular profiling for advanced and metastatic disease [4]. Other emerging biomarkers which are under investigation are *PIK3CA* mutations, high level *MET* amplification, *ERBB2* mutation, and Tumor Mutational Burden (TMB) [4,5].

Current studies also showed that lung adenocarcinomas are not only related to oncogenic addictions (75%) but also non-oncogenic addictions or oncogene negative mutations (25%), which are tumor suppressor genes through loss-of-function mutations and deletions [3]. The current booming players of the tumor suppressor genes category are *TP53*, *STK11*, *KEAP1*, *RB1*, *SMARCA4*, *BRCA1/2*, *NF1*, and *CDKN2A*. Treatments related to these gene alterations are still being formulated [3].

In this context, we highlight our review on the physiopathology of the SWItch/Sucrose Non-Fermenting (SWI/SNF) complex-regulated *SMARCA4* gene, the clinicopathological features of SMARCA4-deficient thoracic cancers, the immunohistochemical detection method in SMARCA4-deficient NSCLC, the necessity of molecular testing in SMARCA4-deficient NSCLC, and the potential therapeutic strategies in SMARCA4-deficient NSCLC.

Prognostic and predictive management issues in SMARCA4-deficient NSCLC. *SMARCA4* gene mutation is reported to encompass 12% of non-oncogenic addicted lung adenocarcinomas and 5% are co-altered among oncogenic-driven lung adenocarcinomas [6,7]. *SMARCA4*-deficient NSCLCs with co-alterations are known to be unfavorable and with almost 50% of these tumors harboring biallelic truncating *SMARCA4* alterations, which causes highly negative effects in the patient, leading to a poor survival outcome [6,7]. The loss of *SMARCA4* function through gene inactivation can be detected through BRG1 immunohistochemistry testing and the mutation can be identified by sequencing of the entire *SMARCA4* gene. Treatment alternatives are yet to be confirmed, although the positive role for platinum-based chemotherapy and anti-PD-L1 therapy have been identified with the observation of the chemosensitive property of the *SMARCA4*-deficient tumors and the high TMB status, respectively.

## 2. SWI/SNF Complex

The double stranded DNA in a eukaryotic cell is formed by three billion base pairs that are condensed into a nucleus, which is the hub of gene expression and transcription [8]. These double stranded DNAs are distributed among 22 pairs of autosomes and 2 sex chromosomes. In the cell nucleus, there are dense areas and loose areas which represent coiled unreadable parts and uncoiled readable parts of the DNA, respectively. Structurally, the DNA strands are negatively charged (phosphate group) and the strands coil around regularly spaced nucleosomes which are composed of 8–9 positively charged histones. This coiling due to the positive-negative charge attraction around nucleosomes is necessary to produce supercoils, which leads to the compaction of the DNA stands to form a chromosome [8].

**Figure 1:**
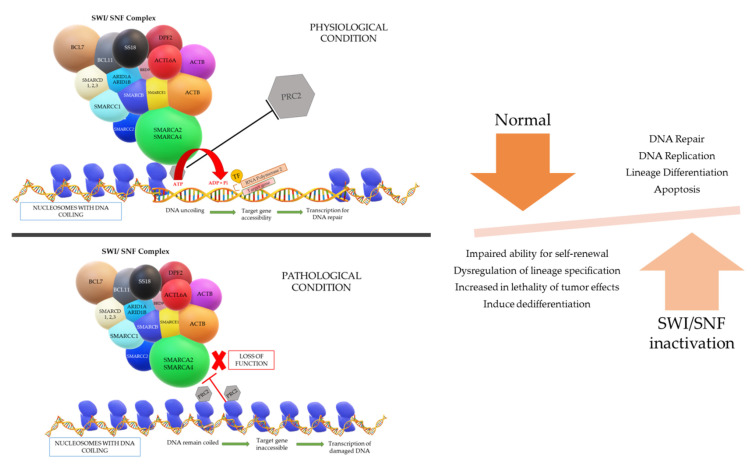
The loss of function of the SMARCA2/A4 catalytic subunit within the SWI/SNF complex due to the inactivation of the *SMARCA4* gene will lead to an increase in DNA damage functionality as PRC2 will continue to bind to the nucleosome and the target gene for DNA repair will be inaccessible as the DNA remains coiled. This will promote the malignant behavior of cancer cells.

A gene is present in a segment of non-repetitive DNA in a chromosome that codes for a specific protein which operates a specific function in the cell physiology. The gene segment present in the uncoiled regions is easily transcribed; however, the coiled regions are not accessible by RNA polymerase and transcription factors unless the coil is loosened. The accessibility of the DNA necessitates an entity called chromatin remodeling complex (CRC), which can change the nucleosome architecture (Figure 1). The CRCs are made up of 12–15 subunits or domains including a core ATPase domain, which is required to create energy by ATP hydrolysis in order to regulate the nucleosome remodeling activity and many other specialized functions [8].

There are four known types of CRCs, specifically the SWI/SNF, ISWI (Imitation SWItch), INO80 (Inositol 80), and Mi/CHD (Chromodomain Helicase DNA-binding) families, which share common functions and also have their own unique functions, but what is known is that the ATPase subunits are commonly shared among the families to conduct their function [8,9]. The SWI/SNF complex was first discovered in yeast in 1984 [10]. The findings also showed that there is a conservation in homology of several features in terms of structure and function of the remodeler throughout the mammalian evolution from *Saccharomyces cerevisiae*, *Drosophila melanogaster*, and mouse to humans [11,12,13].

The physiological process of the SWI/SNF complex is such that when there is a gene that requires to be expressed for repair of DNA damage, the complex will be admitted near the region of damage and subsequently binds to the nucleosome if the DNA region required for transcription is coiled tightly around a particular nucleosome. The SWI/SNF complex will then reposition or remodel the binding nucleosome to promote accessibility of the DNA region for the transcription of the target gene. However, energy is vital for the repositioning and remodeling of nucleosomes, which is fueled by the hydrolysis of ATP in the ATPase domain of the SWI/SNF complex [8,11,12].

The different models that can occur to uncoil the DNA are: (i) repositioning the nucleosome through a sliding mechanism and this widens the space between nucleosomes to give access to the target DNA region, (ii) histones can be evicted by the CRCs and the DNA will be uncoiled due to omission of the nucleosome, and (iii) replacement of a histone by a different histone variant causes reduction in the positive charge of the nucleosome and lessens the hold on the DNA coil. After the loosening of the DNA coil, RNA polymerase and transcription factors can bind to the target gene site and successfully transcribe for DNA repair, replication, lineage differentiation, apoptosis, etc. Another aspect in the loosening of the DNA coil that is understood is the eviction of the polycomb repressive complex 2 (PRC2) by a direct counteraction with the SWI/SNF complex to bind on nucleosomes. This PRC2 complex has the opposite function to the SWI/SNF complex, in which it is responsible for chromatin compaction. Once the “switch” occurs, chromatin de-compaction, DNA transcription, and gene expression come into play [8,11,12].

In summary, the SWI/SNF complex is a powerful regulator for DNA transcription and various types of DNA repair, with a master role in cell cycle progression, cell fate acquisition and cell differentiation in a time- and tissue-specific manner. The complex function is also essential in transcriptional cascade necessary for embryogenesis, playing a role in aspects of brain, eye, vascular system, and heart development. It is the source of tumor suppressor genes regulation, and as mentioned before, it has an antagonistic action against the Polycomb group of complexes [12].

## 3. BAF/PBAF Complexes, BRG1 Protein Subunit, and Its Impact on Cancer

In mammals, the SWI/SNF complex is known as the BAF (BRG-/BRM-associated factor) and PBAF (Polybromo-associated BAF complexes). The BAF and PBAF complexes each contain 10–15 additional subunits, and together they are the products of 29 genes that are responsible for a wide variety of functions of the complexes. These complexes are as such tissue-specific and site-specific in the human body; therefore, they share some similar subunits while others are distinctive [12,14].

Only two subunits possess a catalytic activity among the subunits identified and these are mutually exclusive in both types of complexes. They are none other than the previously described catalytic ATP-dependent helicases, which are encoded by *SMARCA2* and *SMARCA4* genes, important to produce energy to facilitate the ATP-dependent functions of the SWI/SNF complex, labeled as Brahma (BRM) and Brahma-related gene-1 (BRG1) protein-based subunits, respectively. Both the SMARCA2 (SWI/SNF-related, matrix-associated, actin-dependent regulator of chromatin, subfamily A, member 2) and *SMARCA4* (SWI/SNF-related, matrix-associated, actin-dependent regulator of chromatin, subfamily A, member 4) proteins are composed of six domains: a QLQ domain, a proline-rich domain, a small helicase/SANT-associated domain, a DNA-dependent ATPase domain (DExDc and HELICc), a retinoblastoma (RB)-binding domain (LxCxE), and a Bromo domain. The binding and stability of the SWI/SNF complex on the DNA is regulated by the interaction between the Bromo domain and the modulation of H3K27 acetylated level of the histone of the nucleosome. Studies show an increased H3K27 acetylation level of lineage-specific genes upon BRG1 knockdown, which indicates H3K27 to be an important marker to differentiate a functioning from suppressed gene loci of human embryonic stem cells (hESCs). The separation of the double stranded DNA is catalyzed with the aid of ATP hydrolyzation by the helicase and DExDc domains [10,14,15]. The BAF complex in mammalians has multitudes of functions in development, and regulation of biological processes. These include embryonic stem-cell renewal, neural differentiation, cardiac development, regulating learning and memory, and regulating proliferation as opposed to differentiation. The complex is frequently involved in cancer development due to gene mutation [11,15]. Hence, multiple studies have revealed that approximately 20% of all human cancers are an effect in the mutation of the BAF enzyme subunits and proteins that are associated with these subunits. Nevertheless, it is also noted that this frequency may be higher [16,17,18,19].

The first SWI/SNF-associated cancer was identified in 1990s, which was a rare, highly aggressive cancer that hit young children known as Rhabdoid tumor, which has a mutation in the gene encoding the *SMARCB1* subunit. It was only recently that a high prevalence in SWI/SNF subunit mutations was observed in many types of cancer by the advent of cancer genome sequence studies [20]. A number of cancers have been known to be the product of loss or mutation in *BRG1*, which includes lung cancer, small cell carcinoma of the ovary-hypercalcemic type, medulloblastoma, Burkitt’s lymphoma, and others, as the mutation of each subunit promotes oncogenesis [16,20]. It is also identified that gene-specific promoter recruitments are led by the coherence of various nuclear receptors, namely estrogen, progesterone, corticoids, retinoic acid, and vitamin D3 receptors with specific SWI/SNF complex components [21]. The *SMARCA4* gene is located in the chromosome 19p13.2 encoding for BRG1 protein and loss of function mutations to this gene alone can lead to the formation of small cell carcinoma of the ovary-hypercalcemic type, uterine sarcomas, sinonasal undifferentiated carcinomas, and gastrointestinal tract pleomorphic carcinomas. However, lung carcinomas, such as NSCLC, primary adenocarcinomas, and squamous cell carcinomas, are represented by a combination of an epigenetic silencing mutation of the *SMARCA4* gene and *SMARCA2* gene, which are primarily located at the chromosome 9p24.3, which encodes the BRM protein. Other tumors that arise from this combination are thoracic undifferentiated sarcomas, undifferentiated high-grade endometrial carcinomas, gastrointestinal tract pleomorphic carcinomas, and undifferentiated/anaplastic renal cell carcinomas with rhabdoid cells [9].

The mechanisms by which cancer is contributed by SWI/SNF mutations are largely unknown and are still being investigated. At this time point, it is understood that mutations in the subunits likely contribute to cancer by affecting the regulation in the transcriptional pathways that are involved in control of proliferation and fate specification [20]. Impaired ability for self-renewal and dysregulation of lineage specification from hESCs are understood to be led by *BRG1* depletion [15]. Deeper understanding is critical, in the hope that it will potentially support the effort in the discovery of targeted therapy.

## 4. *SMARCA4* Gene Mutation and Its Heterogeneity

In general, *SMARCA4* has been hypothesized as a tumor suppressor gene in primary lung tumors and it is required in growth arrest and cell senescence, by regulating gene expression; hence, it is a critical entity for cancer progression [14,22,23]. The *SMARCA4* gene undergoes an inactivating type of mutation to the gene that leads to a transcription error, causing loss of function of the subunit in the BAF complex and loss of BRG1 protein expression in the nuclei of the tumor cells. There are 120 unique *SMARCA4* mutations that have been reported in the literature. A total of 30–35% of NSCLC cell lines are frequently known to have biallelic *BRG1* inactivation and a subset of primary lung tumors are known to have an absence of BRG1 protein caused by *BRG1* gene mutation [21,24]. A total of 49–50% of the mutations are generally known to be missense mutations, which do not cause as much effect as compared to a total loss of function or even a loss of BRG1 expression. However, 41% of the mutations are part of truncating mutations, namely nonsense, frameshift, insertion, deletion, and splice site mutations, which have a deleterious effect in the loss of function of the subunit. Nevertheless, a few studies also showed that for truncating mutations to take full somatic effect, and to be visualized as a loss in immunostaining of the BRG1 antibody on the tumor cells, the mutation should be biallelic [6,7,11,14]. Additional studies demonstrated that there are multiple point mutations scattered along the *BRG1* gene-coding regions in the mutated case, represented by a complete loss of BRG1 expression. However, an arrest in the cell cycle and flat cell morphology can be re-created in a homogenously deleted or mutated *BRG1* inactivated case, by re-introduction of BRG1 [23].

The dual inactivation of *SMARCA4*/*BRG1* and *SMARCA2*/*BRM* genes of the ATPases in SWI/SNF chromatin remodeling complexes not only creates an epigenetic dysregulation and an increase in the lethality of the tumor effects, it also induces dedifferentiation of a normal to low grade tumor into a tumor with or without rhabdoid features that is graded as highly aggressive. Recent studies reported that the absence of *SMARCA2* is equally necessary for the survival of *SMARCA4*-deficient tumor cells. Many well-established studies have primarily illustrated this dual loss in the small cell carcinoma of the ovary-hypercalcemic type (SCCOHT). The discovery of *SMARCA4* and *SMARCA2* function restoration by the potential treatment with histone deacetylase (HDAC) inhibitor, Tricostatin A for SCCOHTs was strategized by understanding that the growth of SCCOHT cell lines were inhibited by the re-expression of *SMARCA4* and *A2*, as *SMARCA2* loss is caused by the absence of mRNA expression rather than a gene mutation, which is expressed by immunohistochemical studies (IHC) as a non-mutational silencing. Therefore, this can be easily restored by inhibiting histone deacetylation or histone modifiers or methylating DNA. Similar non-mutational *SMARCA2* silencing has been also found in concomitant deficiency with *SMARCB1* in malignant rhabdoid tumors. In view of this shared genetic properties of rhabdoid tumors, it is as much again required for the exploration on HDAC inhibitors [25].

## 5. Clinicopathological Features of *SMARCA4*-Deficient Thoracic Cancers

The mutational inactivation leading to *SMARCA4* deficiency, partly similar histomorphological features and the loss of anti-BRG1 IHC expression with predominantly having transcriptomic similarities to BAF-deficient sarcomas, these *SMARCA4*-deficient thoracic tumors were initially under the umbrella of *SMARCA4*-deficient thoracic sarcomas (DTS) [14]. However, the latest WHO classification of thoracic tumors has separated this entity into *SMARCA4*-deficient undifferentiated thoracic tumors (DUT) and *SMARCA4*-deficient non-small cell lung carcinoma (*SMARCA4*-dNSCLC) [26]. Morphological overlaps are seen between these two entities; however, the exact link between the two is still being studied.

The thoracic *SMARCA4*-DUT is known to localize mainly at the mediastinum, and it can arise at the pulmonary hilum, lung, and/or pleura with or without chest wall invasion. Young to middle aged adults (27–90 years) with preponderance to the male sex and a smoking history are commonly affected [27]. An average of 10% of never smokers can also be victims of this aggressive high-grade tumor. Imaging will reveal the tumor presentation as an ill-defined invasive mediastinal mass which compresses adjacent structures and with metastatic lesions at presentation. It rarely occurs as a small mass in a primary lung tumor. The bone, lymph nodes, adrenal glands, abdominal cavity, and brain are the frequent metastatic sites. Hence, patient presenting symptoms are uncommonly dyspnea, pain, superior vena cava symptoms, weight loss, and other symptoms related to the metastasis. These tumors have been studied to have high tumor mutational burden, mainly a biallelic inactivation by nonsense and frameshift mutational tumorigenesis, less commonly missense mutation, splice-site mutation, deletion with loss of heterozygosity or deletion alone, or with a second mutation. Co-inactivation of TP53 and a subset of NF1 mutations are not unfamiliar to this tumor. Its transcriptional profile is different from *SMARCA4*-dNSCLC but is genomically closely related to the conventional NSCLC. It can arise as a combined tumor with conventional NSCLC with different phenotypic and clinical significance (5%). Patients succumb to its pronounced disease course within months. The median overall survival data available is 5 to 7 months with a 12.5% 2-year survival rate [14].

**Figure 2:**
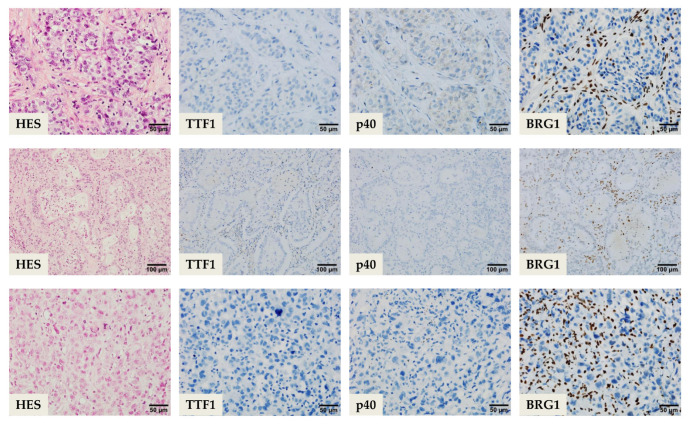
Morphologic and immunohistochemical features of thoracic SMARCA4-deficient tumors. The three main patterns are shown. Upper row, poorly differentiated solid pattern; middle row, the mucinous glandular pattern; lower row, the rhabdoid/undifferentiated sarcomatoid pattern, demonstrating a lack of expression of TTF1 and p40. Immunohistochemical loss of SMARCA4 (BRG1) expression in tumor cells, with internal positive control staining in inflammatory and stromal cells. Scale bars are presented.

*SMARCA4*-dNSCLC is a smoking-associated undifferentiated or dedifferentiated aggressive lung carcinoma. It is predominant among young men with a median age of 63 years and highly preponderant to pleural and vascular invasions [19,28]. The histomorphology of the tumor briefly appears to be a poorly differentiated and as a vaguely defined large cell carcinoma [9]. It is observed to have three main patterns (Figure 2):

(i) A poorly differentiated solid adenocarcinoma pattern with a prominent inflammatory background. The epithelial cells are large and cohesively arranged in compact solid nests and sheets with well-defined cellular borders and characteristic eosinophilic to clear cytoplasm. Occasionally, the cytoplasm contains hyaline secretory globules of various sizes. The poorly differentiated areas can be concomitant with classical well- to moderately differentiated grades of NSCLC [19,28];
(ii) A mucinous pattern is composed of minimal to unequivocal glandular patterns with small abortive lumina, scattered bluish secretory spherules mimicking goblet-like cells, and intracellular and occasional extracellular stromal mucin pools. Focal acinar-like, papillary areas and occasional intermingled rhabdoid cells can be identified;
(iii) A rhabdoid/undifferentiated sarcomatoid pattern is characterized by medium- to large-sized polygonal rhabdoid cells with dyscohesive and pseudoalveolar arrangement. The rhabdoid cells display eosinophilic eccentric cytoplasm, and large vesicular nuclei with eosinophilic macronucleoli. The cells can be bi- or multinucleated. The surrounding stroma is usually inflamed and contains prominent areas of necrosis. Some cases can have extensive undifferentiated sarcomatoid areas. Important clues to note are that there is a complete lack in lepidic and squamous patterns in this tumor [19,28].

Matsubara et al. mentioned their hypothetical scheme of the development of lung adenocarcinomas with *BRG1* and *BRM* loss that can be further divided into two types, based on the terminal respiratory unit (TRU) of the lung [24]. The TRU-type is a lung adenocarcinoma with bronchial epithelial phenotype with initial oncogene mutations such as *EGFR*, *KRAS*, and *MET* amplification, *HER2* amplification, and later a second mutation leading to the loss of the BRM protein causing a subsequent poorer differentiation of the tumor. The second type, the non-TRU-type is a lung adenocarcinoma with a mesenchymal-like, more malignant phenotype harboring concomitant BRG1 and BRM protein loss, which then accelerates the tumor to have poor differentiation with epithelial-mesenchymal transition (EMT) and eventually a poorer prognosis as compared to the single loss of BRG1. This hypothesis may explain the diverse morphology patterns that are observed in the *SMARCA4*-dNSCLCs; however, the exact mechanism of the tumor development and EMT with dual loss of BRG1 and BRM as the causative factors is unknown. However, we are familiar that the uncontrolled cellular proliferation and disrupted differentiation of the bronchial epithelial cells are known to be caused by the dual loss. This is well expressed by the loss of Cytokeratin 7 (CK7), Thyroid transcription factor-1 (TTF1) and Mucin1, cell surface-associated (MUC1) IHC staining of the tumor. This understanding may shine a light on a possible new epigenetic therapy that is aimed to restore BRG1 and BRM functions in the treatment of EMT featured lung tumors [24].

## 6. Immunohistochemical Detection Method in *SMARCA4*-Deficient NSCLC

A broad IHC panel is often required to aid the diagnosis of a *SMARCA4*-dNSCLC as the large cell is poorly differentiated and rhabdoid morphology needs consideration of a wide range of differential diagnosis to be excluded as a primary step [29]. The differential diagnosis would include large cell carcinoma, neuroendocrine carcinoma, NUT carcinoma, lymphomas, melanomas, and various other sarcomas, such as malignant rhabdoid tumors, *CIC*-rearranged sarcomas, epithelioid sarcomas, and giant cell tumors [14]. Metastatic carcinomas and other BAF-deficient tumors, besides *SMARCA4* deficiency, also carry similar morphological features and it is a pre-requisite to be factored out [14].

Agaimy et al. suggested a five IHC marker panel consisting of TTF1, CK7, Pan-cytokeratin (PanCK), HepPar1 (Hepatocyte Paraffin 1), and BRG1 antibodies [28]. *SMARCA4*-dNSCLC cases are predominantly (90%) TTF1 and p40 negative, and if they are positive, they usually have a weak to moderate expression. A diffuse and strong CK7 expression is noted in 90% of cases and the cases with rhabdoid pattern may variably show a lack in CK7 and HepPar1, scattered single cell expression of PanCK with strong Epithelial Membrane Antigen (EMA) reactivity. Although commonly seen in *SMARCA4*-DUT, 85% of the *SMARCA4*-dNSCLC with hepatoid differentiation display diffuse strong granular cytoplasmic expression pattern of HepPar1 or they could have only focal but strong expression. A subset of cases can focally express Sal-like protein 4 (SALL4) and Glypican-3; a majority of cases are distinguished from *SMARCA4*-DUT, as it is predominantly SALL4 negative and it can be differentiated from a metastasis of primary liver lesion by the negativity to Glypican-3, Alpha-fetoprotein (AFP) and Arginase-1. A total of 12% of these tumors are also known to have focal to diffuse weak intensity Synaptophysin reactivity; hence, precaution to exclude a large cell neuroendocrine carcinoma should be advocated [28].

**Figure 3:**
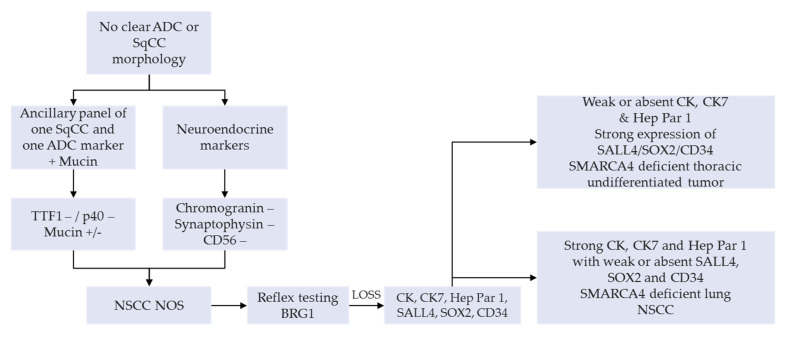
Proposed diagnostic algorithm on small diagnostic samples, including the reflex testing for the anti-BRG1 immunohistochemistry analysis in order to identify the thoracic SMARCA4-deficient undifferentiated tumors. Abbreviations: ADC, adenocarcinoma; NOS, not otherwise specified; NSCC, non-small cell carcinoma; SqCC, squamous cell carcinoma.

A manageable option on small diagnostic samples (e.g., bronchial biopsies and cytology products) or NSCLC-NOS can have a SMARCA4 IHC as a reflex testing, succeeding a negative TTF1, p40 as well as neuroendocrine tumor cell expression. This is then followed by a lengthier panel for the tumor workup (Figure 3) [25,30]. An appealing alternative, subject to clinical validation, would be the use of multiplexed immunohistochemistry combining these diagnostic markers in order to preserve tumor tissue for subsequent IHC or molecular analyses [31,32]. Systematic use of appropriate IHC panels, including anti-BRG1, enables accurate classification of most of the undifferentiated carcinomas, such as carcinomas of unknown primary site, as well as careful preservation of tissues for potential molecular or other ancillary tests [33].

As has been established, the most common *SMARCA4* alterations originate from a biallelic truncating in-frame mutation which leads to the inactivation of the *SMARCA4* gene causing loss of protein function and a complete loss of anti-BRG1 expression. Although various antibody kits and distributors are present in the market, the most commonly used anti-BRG1 antibody is the EPNCIR111A clone (Abcam, Cambridge, UK; dilution 1:100) [18,25,28,30]. Multiple studies have also expressed the need to perform SMARAC2/anti-BRM antibody testing (e.g., anti-SMARCA2 polyclonal antibody HPA029981, Atlas Antibodies, Stockholm, Sweden) in the same setting due to the known deleterious prognostic effect on tumors with a dual loss of the catalytic ATPase subunits. Multiple studies have concluded about 5 to 12%, 5 to 17%, and 10% of primary pulmonary adenocarcinomas with either individual IHC loss of BRG1 or BRM, or concurrent IHC loss, respectively [28,30,34]. Herpel et al. also reported a 30% outcome in human NSCLC cell lines exhibiting concomitant loss of BRG1 and BRM by western blotting [30]. Well-established IHC interpretation of the stains have also been reported in several studies, suggesting that deficient/loss of expression is a label for unequivocal complete absence of the nuclear staining of viable tumor cells (evaluation away from the necrotic areas) or a very weak homogenous but recognizable staining of viable tumor cells as opposed to the stronger expression of the normal background cells giving the impression of reduced expression. Intact expression is observed when the tumor cells show intense nuclear uptake that is as equivalent to the non-neoplastic cells in the background. In addition, a hybrid expression loss may be observed when a subpopulation of tumor cells show loss amidst a population of tumor cells with intact staining. The internal control cells are the stromal fibroblasts, inflammatory cells, vascular endothelial cells, and normal epithelial cells in the background, which exhibits strong nuclear staining. On the contrary, it is deemed not assessable when the internal control cells lack expression [28,30,34]. It has also been reported that tumors with BRM are intact/positive in cases where more than 5% of tumor cells shows nuclear staining and loss/negative in cases where only up to 5% of tumor cells display intact staining. Cytoplasmic staining is not considered in the interpretation of the IHC antibody staining [25].

In the study by Matsubara et al., which worked on 15 lung cancer cell lines and 11 out of 93 primary lung adenocarcinomas, showed cases of BRG1 mutation, including 16 cases with concomitant loss of BRM expression, which frequently showed negative to E-cadherin and positive to Vimentin expression supporting the epithelial-mesenchymal transition of these types of tumors [24,35]. Similar findings were identified in a subset of cases with dual loss in another analysis on a sample size of 442 cases of primary adenocarcinomas. The authors also emphasized that the loss of BRG1 and BRM is co-related to the absence of lepidic growth pattern and represented by a strong immunoreactivity to BRG1 and BRM antibodies [24,35]. The study reported that the well- and moderately differentiated adenocarcinoma areas and some cases with papillary or acinar patterns have intact BRG1 and BRM expression, whereas poorly differentiated or solid adenocarcinoma with mucin areas has loss of BRG1 and loss/weak expression of BRM [24].

## 7. Necessity of Molecular Testing in *SMARCA4*-Deficient NSCLC

Previous Next Generation Sequencing (NGS) studies have identified that a mutation in the *SMARCA4* gene does not necessarily result in loss of protein expression. As mentioned in the chapters above, it is necessary to recognize that missense mutations and single allelic representations do not give rise to a loss in anti-BRG1 expression as there is no functional defect in the protein. In order for a deleterious mutation that exhibits a possible loss of protein expression, a biallelic inactivation through nonsense or in-frame deletions is imperative [6,28]. Conversely, in some cases, IHC can be uncertain and molecular testing is advised in such instances [14].

The SMARCA4 and SMARCA2 ATPases belong to the SNF2 family of DNA-dependent ATPases related to DEXDc and Helicase C domains; nevertheless, it is also known that the BRG1 and BRM are multidomain proteins [6,16]. This is further supported by evaluations showing that the mutations largely involve the entire span of the *SMARCA4* gene rather than hotspot clustering within the gene [6,28,36]. Studies identified nine *SMARCA4* mutations widely distributed along the *SMARCA4* gene, which includes Glu1023* and Glu1346del mutations, as well as a common missense mutation G1232S/V in lung adenocarcinoma patients [7,19]. There are several NGS platforms and panels used by various studies (e.g., (i) FoundationOne assay, 324 gene panel—gene expression microarray technique, using HiSeq 2000 instrument to perform whole exome sequencing and tumor mutational burden calculation; (ii) 160 gene NGS panel with full coding sequence of *SMARCA4* gene using MiSeq instrument with MiSeq Reagent Kit V3 (Illumina) and CLC Genomics Workbench Analysis Software (Qiagen); and (iii) Oncomine Cancer Research Panel (Oncomine Comprehensive Assay v3) covering 161 genes using Ion Chef and Ion S5 Systems (ThermoScientific) [6,7,19,28,35]). More understanding on the *SMARCA4*-dNSCLC shed some light on its correlation and effects on co-existence with oncogenic drivers and other tumor suppressor genes as well as possible biomarkers and immune check point inhibitors to determine optimal treatment options for metastatic tumors. In view of this, tumor tissue may be limited to a long list of investigations, and incorporating multiple IHC marker panels may not be practical; therefore, identification of *SMARCA4* among the panel of other genetic markers of interest through NGS-based strategies may be more ideal [6]. Keeping in mind that sequencing is not a dying necessity to make the diagnosis of *SMARCA4*-dNSCLC, as complete IHC loss is sufficient, it does help clarify the cases of reduced expression of BRG1, in which mutation detection is very much reliant on the detection method used [6].

The uniform lack of actionable gene alterations of conventional lung adenocarcinomas; namely, *EGFR* mutation, *EML4-ALK* rearrangement, and *ROS1* rearrangement are common knowledge with regards to the *SMARCA4*-dNSCLC tumors [28]. The loss of BRG1 is specific to the progression of an *EFGR* wild-type NSCLC tumor as compared to an *EGFR* mutant tumor even so, the pathogenesis of it is still being investigated [28,30].

Wu et al. identified that tumor protein 53 (TP53) regulation is necessary for the proliferation of HeLa cells by BRG1 function. However, there is no clear research has been done on the BRG1- and TP53-mediated transcription and inactivation of this protein in *SMARCA4*-deficient tumors [16].

As mentioned, BRG1 plays an important role in growth control, and this is done by the interaction with the tumor suppressor Retinoblastoma protein (pRb). The pRb is wild type in major BRG1 mutant cell lines. Hence, some reciprocity between the BRG1 and RB gene mutations are seen, such as BRG1 protein is necessary for RB-induced growth inhibition. The pRB interaction in turn disables the cyclin-dependent kinase inhibitor 2A (p16) to arrest a BRG1 cell line [23,34].

In justification of the number of genes and proteins that are required to be tested for the management of the *SMARCA4*-dNSCLC, and as the lack of tissue material for testing is a major issue in the field of laboratory medicine, NGS-based panel testing is an appropriate option.

## 8. Potential Therapeutic Strategies in *SMARCA4*-Deficient NSCLC

A common approach towards NSCLC beyond the early-stage treatment would be radical surgery, followed by platinum-based chemotherapy and/or radiotherapy, but it is the dire need for new therapeutic approaches which has brought us to the current era of personalized medicine and targeted therapy. *SMARCA4*–dNSCLC is not druggable at this time point, but several pre-clinical studies have shown some distinctive drug sensitivity, though more efforts are needed for confirmatory beneficial findings [28]. Since SCCOHT has been studied longer than *SMARCA4*–dNSCLC, potential treatment strategies have been worked out to create a paradigm shift in the epigenetic dysregulation of the prior tumor. The treatment possibilities are developed to target agents that regulate epigenomes, such as histone deacetylase (HDAC) inhibitors, histone modifiers, or histone demethylases [37]. This also raises the possibility to avoid the loss of SMARCA2 in SCCOHT using inhibitors of ATPase/Bromodomain, since the cell line survival is dependent on the *SMARCA2* loss in a *SMARCA4*-deficient tumor. The hope is to eventually be able to predict the response to drugs that target epigenetic modifications. Preliminary studies on the HDAC inhibitors do support this motion [25].

The defect is also in the failure to activate the genes that promote or maintain terminal differentiation. For example, tumors with rhabdoid features are aggressive and of high grade due to the dedifferentiation from a normal cell or low-grade tumor, induced by dual deficiency in *SMARCA4* and *SMARCA2*, but an effective therapy to improve the outcome is still being investigated. SWI/SNF complex and Myc proto-oncogene (c-myc) have a mutually exclusive functional relationship, as c-myc physically interacts with the chromatin remodeling complex, INI1 and BRG1. As c-myc is an oncogene and BRG1 is a tumor suppressor gene, the interaction between both is an antagonistic relationship. In the study by Romero et al. on the antagonistic effect of BRG1 on c-myc activity and the promotion of cell differentiation in human cancer it was suggested that the upregulation of lung-specific transcripts and restoration of gene expression signature of normal lung can be done by re-expression of BRG1 [21]. They found the unresponsiveness towards retinoic acid or glucocorticoids of a variety of cancer cell line lacking BRG1 can be reversed by restoration of BRG1 function. This antagonistic effect is also evident in primary tumors. BRG1 inactivation leads to the dysregulation of c-myc activity in the cancer cells, preventing appropriate gene expression control due to the no response towards nuclear receptors such as estrogens, progesterons, corticoids, retinoic acid, and vitamin D3 and sustainability of undifferentiated gene expression programs by cancer cells, thereby promoting cell growth and maintaining an undifferentiated status. However, the BRG1 restoration mechanism has been deduced through nude mice implantation of the lung cancer cells, which show a decrease in c-myc activity and significant dampening of cancer cell progression and invasion, and this also illustrates its tumor suppressor property [21]. Hence, a drug that supplements BRG1 or countermand BRG1 inactivation needs to be developed.

A large area of research in *SMARCA4*-dNSCLC tumor treatment is being dedicated towards tumor suppressor activity of bromodomain and extra-terminal motif protein inhibitors (BETi) across several cancer types. However, the studies still fail to find predictive biomarkers to identify patients who will benefit from this treatment. The study models showed the development of resistance to BETi upon reactivation of *SMARCA4* or SMARCA2, but a knockdown of *SMARCA4* sensitized the resistant cells to BETi. Therefore, BETi hypersensitivity is based on *SMARCA4* or *SMARCA2* loss, signifying *SMARCA4* as a possible predictive biomarker for a case eligibility to receive BETi therapy. These findings reveal that *SMARCA4/A2* deficient aggressive cancers may potentially have a therapeutic strategy with the emergence of BETi. To date, BETi therapy has been used in phase 1 trials for multiple myeloma, acute leukemia, and NUT midline carcinoma, which was reported to have complete responses but not to expectations, as the trial has not succeeded in detecting biomarkers to predict patient responses; therefore, subsequent long-duration studies are required [38]. 

Studies have increasingly proven that *SMARCA4*-dNSCLC is chemo-sensitive to platinum-based chemotherapy [28]. A gene expression study with microarray demonstrated that reduced *SMARCA4* expression is associated with worse prognosis and poor overall survival; however, this was shown to be remedied in patients with low *SMARCA4* expression receiving adjuvant cisplatin or a Vinorelbine chemotherapy agent, which improves the 5-year disease-specific survival. In this study, the authors concluded that the increased sensitivity to platinum-based chemotherapy in NSCLC can be predicted through the low expression of *SMARCA4*/BRG1 acting as a novel biomarker and it depicts the efficacy of the drugs independent of age, stage, and histology in resectable NSCLCs; this, in turn, also encourages pathologists and oncologists on the importance of recognizing the clinicopathological features of *SMARCA4*-dNSCLC [30,39]. Despite the advent of targeted therapy, chemotherapy is still used for the treatment of early and late-stage lung cancer to prolong overall survival; however, the extension is rather small, while patients are exposed to the deleterious toxic effects of the chemotherapeutic agents. Although biomarkers for targeted therapy are well-established, the biomarker to measure the responders from non-responders of chemotherapy remains limited despite numerous attempts to evaluate the impact produced from tumor suppressors [39]. Both *SMARCA4* and SMARCA2, as predictive biomarkers for cisplatin-based chemotherapy, were first reported using NSCLC patient specimens by Bell et al. [39]. The adjuvant cisplatin-based chemotherapy in NSCLC patients can also reduce the risk of recurrence post-complete resection in unselected stages IB, II and IIIA patients; however, not all will benefit from the reduction while no one is spared from the effects of toxicity. Defects in DNA repair is primarily the mechanism for an increase in cisplatin sensitivity in lung cancers with BRG1 or BRM knockdown. Therefore, identifying some biomarkers can accurately select patients with NSCLC or other cancers that are eligible for platinum-based regimens through a panel of molecular markers comprised of epigenetic regulators and DNA repair genes. The multipanel approach is best to consider, as there is evidence that in some cases, despite the lack of expression, there is no mutation detected in *SMARCA4/A2*. Although there is no limitation in age, Bell et al. incidentally also found excellent overall advantage with platinum-based chemotherapy in patients of 65 years and older bearing low levels of BRG1 expression. Overall, although we are convinced that *SMARCA4*-dNSCLC is chemo-sensitive to platinum-based therapy, the potential to serve as companion diagnostics and the method of detection warrants further research for *SMARCA4* as a predictive biomarker for cisplatin and other DNA repair targeted therapy in NSCLCs [39].

*SMARCA4*-deficient tumors have been demonstrated to have increased lymphocytes, macrophages, and multinucleated giant cells in the background, which histologically corresponds to the increased in inflammatory cells in the surrounding tumor stroma, and the SWI/SNF complex has also been identified to modulate the immune system. This brings forward many research and clinical trials evaluating the role of programmed death-ligand 1 (PD-L1) regulation by BRG1, which concludes to a mixed outcome. Some studies conclude that there is an increase in PD-L1 ≥ 1% positivity in NSCLC cases of BAF loss versus an intact BAF, meanwhile no difference can be seen in the BAF status for cases with PD-L1 > 50% positive cells [19,35]. Concurrently, other studies have shown 15% out of the 46% of BRG1 loss NSCLC cases exhibit ≥ 50% of the total positive PD-L1 expression. A recent multicohort study effectively reported an efficacy of up to 30% in NSCLCs treated with immune check point inhibitors (ICI). In terms of tumor mutational burden (TMB), it is an undisputed result that TMB is increased in BAF loss tumors as compared to intact BAF tumors. In conclusion, NSCLC with loss of the BAF complex is associated with a PD-L1-positive status in general and an increased TMB [6]. Several success stories on using ICI in the management of advanced *SMARCA4*-dNSCLC and *SMARCA4*-deficient malignant rhabdoid tumor-like tumors have been reported. In the case report by Naito et al., Nivolumab was used as a fourth line of treatment in a 43-year-old man with a history of smoking diagnosed to have *SMARCA4*-dNSCLC with multiple lung metastasis and recurrence after standard chemotherapy regimens [19]. Whole exome sequencing revealed a high TMB despite lack of PD-L1 expression. Disease control maintained for more than 14 months after continuous tumor shrinkage, as evidenced by chest CT, through 22 doses of Nivolumab [19]. Acase report on the long-lasting response of Pembrolizumab showed that a 58-year-old woman diagnosed with a *SMARCA4*-deficient malignant rhabdoid tumor-like tumor, characterized by dual loss of SMARCA4 and SMARCA2 on IHC had remarkable symptomatic recovery from the treatment. She had a 72% partial response after 11 months of treatment, despite having very low TMB and only scattered immune cell positivity for PD-L1 [40]. Pembrolizumab as a first-line treatment was also effective in drastic tumor growth suppression with just a single dose, giving rise to an asymptomatic and clinical partial response with no adverse effects in a 69-year-old female with mediastinal *SMARCA4*-DTS with metastasis and a positive tumor cell PD-L1 status of > 60% [41]. There are also reports on four cases of SCCOHT with major responses to ICI therapy. In view of all these reported cases, there is a high possibility that ICI therapy can be considered as a treatment option for *SMARCA4*-deficient tumors with or without a PD-L1 IHC response by efficiently reducing tumor burden both clinically and symptomatically. However, determining the anti-PD-L1 blockade sensitivity mechanism is still ongoing [41]. Anti-tumor activity in pre-clinical models of *SMARCA4*-deficient tumors have recently been identified in CDK4/6, AURKA, ATR, and EZH2 inhibition; hence, promising results await through future trials on the use of these agents alone or in combination with ICIs [42].

## 9. Prognostic and Predictive Management Issues in *SMARCA4*-Deficient NSCLC

There are several issues related to *SMARCA4* co-mutation with oncogenes and tumor suppressor genes as well as the correlation with therapeutic options that influence the prognosis and predictive factors in the management of *SMARCA4*-dNSCLC. It has been established that *SMARCA4*-dNSCLC is independently a highly aggressive tumor with poor patient survival outcome, regardless of the TNM stage [6,9,19,28]. In a recent genomic landscape study of *SMARCA4* alteration and its association with the outcome involving 4813 lung cancer patients, *SMARCA4*-mutated NSCLC was divided into two clinically relevant genomic classes [42]. In total, 8% of the cases showed a loss of protein expression that had truncating, fusion, and homogenous deletion mutations that were labeled as class 1 tumors and class 2 tumors had intact protein associated with missense and non-truncating mutations. The classes have different protein expressions with distinct prognosis, and subsequently, have different treatment implications. In cases of class 1 alteration, further shortened overall survival is seen in a metastatic *SMARCA4*-dNSCLC. However, these class 1 tumors are the best responders for ICI treatment with improved outcomes. The study identified that both classes more commonly co-mutated with *TP53*, *KRAS*, *STK11*, and *KEAP1* mutations as compared to these co-mutations in a *SMARCA4* wild-type tumor, and to a lesser extent, it is not uncommon to have co-alterations with other oncogene drivers, i.e., *EFGR* mutation. This has differential responses to immunotherapy and its effect on long-term survival. The study identified that *SMARCA4* deficiency associated with *KRAS* co-mutation has worse prognosis than solitary *SMARCA4* mutation, especially in Class 1. The Class 1 alteration with protein loss seems to be the strongest independent factor for unfavorable patient prognosis but with an upside of the best ICI treatment outcome. Patients with *KRAS*-mutant tumors co-harboring *STK11* and *KEAP1* alterations show dampened benefits to immunotherapy and poorer prognosis, and an additive impact of shortened overall survival is also seen with a supplementary *SMARCA4* mutation. A function compromise, despite the intact expression in Class 2 *SMARCA4* alterations, is evident through an overall poor prognosis of such tumors relative to the *SMARCA4* wild-type genotype. The mechanism for this compromise was worked up to identify that the missense mutation makes changes in the proto-oncogene expression in c-myc and its target genes when it modifies the open chromatin landscape. Despite the poor outcomes associated to co-mutations, *SMARCA4*-mutant lung cancers are, in general, more sensitive to immunotherapy and may have an advantageous therapeutic option in the future [42].

The special relationship carried by *KRAS*-driven lung cancers with *SMARCA4* loss was studied by some research groups [7,43,44]. As is already known, *KRAS* is a key oncogenic driver in lung adenocarcinomas, which is frequently observed in ever smokers and it is associated with a high TMB; hence, a subset of cases which carry other mutations may show a better response to ICI therapy. It was also eminent that *SMARCA4* plays a role in growth and tumorigenicity of *KRAS*-driven lung cancers by supporting the oncogenic transcriptional signaling landscape of the tumor. Distinguishing this pathogenesis supports that treatment should be aimed directly towards the SWI/SNF complexes in lung adenocarcinomas and other malignancies related to *SMARCA4* mutation. Findings show that *KRAS*-independent lung adenocarcinomas harbor higher rates of *SMARCA4* inactivation as compared to *KRAS*-addicted lung adenocarcinomas. It is important for *SMARCA4* to be intact in oncogene-driven lung adenocarcinomas, as loss of *SMARCA4* dampens the *KRAS* signaling in a *KRAS*-addicted cancer and, subsequently, impairs proliferation of the tumor cells. Hence, such co-mutated tumors consistently have small bland nuclei of lower grade characteristics and lower overall tumor Ki67 proliferative index. Therefore, it is acknowledged that a precursor lesion with *KRAS* mutation progresses to malignancy through an acceleration of an intact *SMARCA4* gene. This is possible when the *SMARCA4* in a *KRAS*-dependent lung adenocarcinoma is a missense mutation, identified through an intact BRG1 expression or the loss of BRG1 expression caused by a nonsense, frameshift, or splice site mutation in the *SMARCA4* gene in a *KRAS*-independent lung adenocarcinoma [7,43]. In summary, the *SMARCA4*-deficient tumors with a *KRAS* oncogenic activation are associated with a better prognosis, as the tumor will be less progressive, low grade, and with lower tumor burden as compared to a *SMARCA4* intact *KRAS* co-mutated NSCLC. However, in contrast to a *KRAS*-only mutation and a *KRAS* co-mutation with *TP53*, the *SMARCA4*-mutated *KRAS*-driven lung adenocarcinomas display poorer clinical outcome with a shorter overall survival and disease-free survival. On the other hand, a remarkable effect on ICI therapy received by patients with *KRAS* and *TP53* mutation was seen that was compared to *KRAS*-*SMARCA4* mutation or *KRAS* mutation alone, as this co-mutation facilitates T-cell infiltration and augments tumor immunogenicity, resulting in a high PD-L1 expression. This, in fact, further enhances the need to evaluate BRG1 IHC expression and molecular evaluation of co-mutations to predict the responders with NSCLC to immunotherapy [7,43,44]. 

As the poor prognosis is recognized in *SMARCA4*-deficient tumors, the presence of frequent co-mutation with other tumor suppressor genes, such as *STK11* and/or *KEAP1*, further dampens the regrettable overall survival and shortens the progression free survival outcome of the disease. However, the worse prognosis also holds true for NSCLCs with the presence of *STK11* and/or *KEAP1* mutation alone without *SMARCA4* mutation or *SMARCA4/STK11/KEAP1* wild-type in comparison to an independent *SMARCA4*-dNSCLC, despite the treatment with ICI or chemotherapy or both. It was also noted that although the pure *SMARCA4* mutations benefit from ICI therapy, there is no significant difference in the efficacy of treatment with ICI found in NSCLC with *SMARCA4* mutation or *SMARCA4* wild-type, and if a resistance to treatment is recognized, the co-mutation status of *STK11* and/or *KEAP1* is a must to test [44,45].

## 10. Conclusions

*SMARCA4*-deficient tumors can be divided into NSCLC and undifferentiated thoracic tumors. The focus of this review is on *SMARCA4*-dNSCLC, which is prevalent in about 5–10% especially among the poorly differentiated adenocarcinomas. Higher percentages of *SMARCA4*/*BRG1* inactivation are concomitantly involved with *SMARCA2*/*BRM* loss. Both genes encode for the ATPase catalytic subunit in the SWI/SNF chromatin remodeling complex, which plays a role in transcription and repair of DNA, proliferation of the human embryonic stem cells, differentiation of the three germ layers, and many more.

Clinically, the *SMARCA4*-dNSCLC cases involve predominantly males, smokers, and a younger age group between 42 and 93 years old. Histopathological patterns of this tumor consist of three variants, but the predominant pattern is the solid poorly differentiated type that can have intermingled rhabdoid cells, and occasional glandular pattern with abortive small lumina. The cells are arranged in cohesive nests and sheet with characteristic well-defined small to large epithelioid appearance with eosinophilic to clear cytoplasm, eccentrically placed nuclei, and giant cell formation in some. The background stroma shows prominent inflammatory cells. A complete lack in lepidic pattern of tumor cell growth is a clue. The *SMARCA4*-dNSCLC has a highly aggressive behavior with vascular invasion and metastasis to the pleura seen upon presentation in most cases. The prognosis of patients is poor, with short overall survival rates.

The best method to the detection of *SMARCA4* and *SMARCA2* deficiencies is through IHC testing, in which the complete loss of BRG1 nuclear staining signifies the inactivation. The complete loss of expression is evident when there is a biallelic deleterious frame shift, nonsense, indel, or splice site mutation, which are present in about 50% of the cases. As for the sequencing techniques, *SMARCA4* and *SMARCA2* are not hotspot mutations; instead, they have multiple mutations throughout the gene. Hence, a sequencing of the whole gene is required. Besides these specific protein and gene testing, we are familiar that *EGFR*, *ALK*, and *ROS1* are wild-type in *SMARCA4*-dNSCLC, which requires targeted sequencing and in situ hybridization. Although the *SMARCA4*-deficient tumors are prognostically worse on its own, the antagonistic effect with c-myc, *KRAS*, and the agonistic effect of co-mutation with other tumor suppressor genes, such as *TP53*, *pRB*, *STK11*, and *KEAP1*, in relation to worsening prognosis, is also important to detect and to enlighten the extent of overall survival of the patient. It is very important to know the *SMARCA4*, PD-L1, and TMB status, as several studies have proven them to be beneficial predictive markers for the currently researched potential therapeutic agents for *SMARCA4*-dNSCLC and *SMARCA4*-DUT. Therefore, the NGS panels are the most effective way to identify all the required oncogenes and tumor suppressor genes simultaneously, as we routinely require working with limited sample quantities. The possible testing algorithm that may be considered in a TTF1- and p40-negative poorly differentiated NSCLC, is a BRG1 IHC as a second line testing. Subsequently a NGS assay to assess the *EGFR*, *ALK*, *ROS1*, *TP53*, *MYC*, *pRB*, *KRAS*, *STK11*, *KEAP1* and TMB can be made concurrent with the anti-PD-L1 IHC. This will summarize the prognosis on overall survival, disease progression, and predictive therapeutic benefits.

Various treatment alternatives are being investigated. Some are in clinical trial phase and others have been reported to be used on individual patients. In the case of a reversal of the c-myc antagonistic effect on *SMARCA4*-deficient cases, the restoration of BRG1 has revealed reversal effects. The theory of using HDAC inhibitors as histone deacetylates to regulate the epigenome and inhibit tumor cell line survival is also a strong potential that requires further exploration. Improvement in tumor suppressor activity in *SMARCA4*/*A2* loss cases with BETi inhibition therapy has gained some interest which still needs more scrutiny. However, at present, the highly positive treatment options are platinum-based chemotherapy (adjuvant cisplatin or Vinorelbine), as *SMARCA4*-deficient tumors are proven to be distinctly chemosensitive, and the promising possibility of anti-PD-L1 therapy in high TMB cases, despite the variable results of anti-PD-L1 IHC with almost 72% partial response and 14 months of disease control maintenance in reported cases that were treated with Pembrolizumab and Nivolumab as first-line and fourth-line treatment, respectively. The potential management options for this subset of tumors in the near future are exciting. Consequently, it is necessary for pathologists to consciously identify this entity; in addition, clinicians should be able to counsel their patients on the disease outcome, with updates on the possible treatment options available.

## Data Availability

Not applicable.
